# Draft Genome Sequence of Burkholderia reimsis BE51, a Plant-Associated Bacterium Isolated from Agricultural Rhizosphere

**DOI:** 10.1128/MRA.00978-18

**Published:** 2018-10-04

**Authors:** Qassim Esmaeel, Alaa Issa, Lisa Sanchez, Christophe Clément, Cédric Jacquard, Essaid Ait Barka

**Affiliations:** a Unité de Résistance Induite et Bioprotection des Plantes–EA 4707, UFR Sciences Exactes et Naturelles, University of Reims-Champagne-Ardenne, Reims, France; Georgia Institute of Technology

## Abstract

Burkholderia reimsis BE51, isolated from maize rhizosphere, has a promising biocontrol activity against a set of phytopathogens. Here, we report its draft genome sequence with the aim of providing insight into the potentially produced secondary metabolites and genes related to plant growth-promoting and biocontrol properties.

## ANNOUNCEMENT

The high demand for agricultural crops is increasing and is expected to keep growing in the upcoming decades. Under exposure to different stress conditions, plant growth and development are affected, leading to a significant loss in crop productivity and quality (1). Traditionally, plant diseases are treated by the application of chemical pesticides, which are not always economical or effective. Further, chemical control methods may have unwanted health, safety, and environmental risks leading to an ecological problem, such as the development of resistance in pathogenic races (2). Therefore, the use of plant-associated bacteria that are able to enhance plant performance and increase their tolerance to different stresses has been reported (3, 4). The genus Burkholderia contains different members that have been isolated from different ecological niches (5–9). Different members of Burkholderia have been reported as plant growth-promoting and biocontrol agents, especially those related to the genus Paraburkholderia (10, 11).

The bacterium strain BE51 described here was isolated from maize rhizosphere according to a protocol that was previously described (12). The strain was found to be Gram negative, motile, facultative aerobic, and oxidase positive. The optimum growth conditions are 30°C and pH 7.0 in the presence of 0.5% NaCl. Based on phylogenic analysis, chemical characteristics, and genotypic data, strain BE51 is distinct from previously known species and represents a novel species of the genus Burkholderia, for which the name Burkholderia reimsis BE51 is proposed. BE51 possesses antifungal activities against Fusarium oxysporum, Fusarium poae, Fusarium graminearum, Fusarium culmorum, Botrytis cinerea, and Rhizoctonia solani and has friendly interactions with grapevine.

The BE51 genome was sequenced at MicrobesNG (http://www.microbesng.uk) using Illumina MiSeq and HiSeq 2500 technology platforms, with 2 to 250-bp paired-end reads, and the mean coverage was 140×. For all software, default settings were used. The nearest reference genome, for Burkholderia lata, was determined using Kraken (13), and to assess data quality, reads were mapped to this genome using the Burrows-Wheeler Aligner (BWA) MEM algorithm (http://bio-bwa.sourceforge.net). The reads were assembled by *de novo* assembly using SPAdes (http://cab.spbu.ru/software/spades/). The draft genome sequences, assembled into 182 contigs with an *N*_50_ contig size of 157,936 bp, was estimated at 8,934,495 bp with a G+C content of 66.40%. The gene function prediction was detected using the Rapid Annotations using Subsystems Technology (RAST) server (http://rast.nmpdr.org) (14), followed by an annotation using the SEED database (15), resulting in 89 RNAs and 7,961 coding sequences distributed in 563 subsystems. The draft genome sequence was mined with the aim of screening all potentially produced secondary metabolites (SMs) using antiSMASH (16). *In silico* analysis revealed the presence of 14 putative biosynthetic gene clusters potentially involved in the synthesis of many SMs, including bacteriocin, phenazines, pyrrolnitrin, siderophores, and nonribosomal peptides. In addition, the BE51 genome harbors genes involved in indole acetic acid production, motility, and biofilm production and one gene related to 1-aminocyclopropane-1-carboxylate deaminase. All these features may reflect the biocontrol and plant growth-promoting effects of this bacterium.

### Data availability.

This whole-genome shotgun project has been deposited at GenBank under the accession no. QMFZ00000000. The version described in this paper is the first version, QMFZ01000000. The SRA accession no. is SRP156928.
